# The Impact of Arts Activity on Nursing Staff Well-Being: An Intervention in the Workplace

**DOI:** 10.3390/ijerph13040435

**Published:** 2016-04-19

**Authors:** Simona Karpavičiūtė, Jūratė Macijauskienė

**Affiliations:** 1 Department of Social Sciences and Humanities, Lithuanian University of Health Sciences, Šiaurės pr. 57, LT-49264 Kaunas, Lithuania; 2 Faculty of Nursing, Lithuanian University of Health Sciences, Eivenių g. 4, LT-50161 Kaunas, Lithuania; jurate.macijauskiene@lsmuni.lt

**Keywords:** nursing staff, organizational well-being, occupational stress management, workplace interventions, arts activity, silk painting, mental health and well-being, arts for health

## Abstract

Over 59 million workers are employed in the healthcare sector globally, with a daily risk of being exposed to a complex variety of health and safety hazards. The purpose of this study was to investigate the impact of arts activity on the well-being of nursing staff. During October–December 2014, 115 nursing staff working in a hospital, took part in this study, which lasted for 10 weeks. The intervention group (*n* = 56) took part in silk painting activities once a week. Data was collected using socio-demographic questions, the Warwick-Edinburgh Mental Well-Being Scale, Short Form—36 Health Survey questionnaire, Reeder stress scale, and Multidimensional fatigue inventory (before and after art activities in both groups). Statistical data analysis included descriptive statistics (frequency, percentage, mean, standard deviation), non-parametric statistics analysis (Man Whitney U Test; Wilcoxon signed—ranks test), Fisher’s exact test and reliability analysis (Cronbach’s Alpha). The level of significance was set at *p* ≤ 0.05. In the intervention group, there was a tendency for participation in arts activity having a positive impact on their general health and mental well-being, reducing stress and fatigue, awaking creativity and increasing a sense of community at work. The control group did not show any improvements. Of the intervention group 93% reported enjoyment, with 75% aspiring to continue arts activity in the future. This research suggests that arts activity, as a workplace intervention, can be used to promote nursing staff well-being at work.

## 1. Introduction

Over the past decade interest in subjective well-being has significantly increased in political and academic sectors. Well-being is often regarded as being synonymous with quality of life and happiness [1]. According to the literature, there are three types of well-being: Evaluative subjective well-being (such as people’s overall assessments of their life or domains of their life); Affective subjective well-being (such as positive and negative feelings); Eudaimonic subjective well-being (such as meaning, autonomy, control and connectedness) [2]. 

Mental health and mental well-being are fundamental to the quality of life and productivity of individuals, families, communities and nations, enabling people to experience life as meaningful and to be creative and active citizens [3]. According to Stewart-Brown, Janmohamed [4], mental well-being covers two perspectives: the subjective experience of happiness (the affect) and life satisfaction (the hedonic); and positive psychological functioning, good relationships with others and self-realisation (the eudaimonic). According to Persechino *et al.* [5], stress is the second most frequently reported work-related health problem in Europe. Well-being and stress in the workplace have become a concern for employees, employers, professional bodies and governments [6]. The mental health and well-being of the workforce is a key resource for productivity and innovation in the European Union, thus promotion and implementation of prevention programmes in the workplace is rapidly growing [7]. 

Around 10% of workers are employed in the healthcare sector throughout the European Union and are exposed daily to a complex variety of health and safety hazards [8]. In the academic sector, there is a growing interest in nursing staff well-being at work [9,10,11], work related stress and its negative impact on nursing professionals’ health [12,13,14]. According to research, the stress nursing staff experiences at work is associated with fatigue and professional burnout [15,16,17]. Hospital nurses’ fatigue has been subject to broad attention in the literature because of its immediate impact on patient safety [18]. In this stark context, an exploration of the potential impact of the arts activity on nursing staff well-being is timely and significant.

Today’s renewed focus on humanistic care is leading to resurgence in the knowledge and practice of incorporating the arts into healthcare settings [19,20,21,22]. According to Almalki *et al.* [23], sustaining a healthy work life for nurses is crucial to improve their quality of life, retain current staff at their workplace, enhance performance and productivity, and promote safe nursing care. Perry and co-workers [24], emphasised the need to create workplaces where working practices promote nurses’ health and well-being, or at least are configured to minimise deleterious effects of work related stress on health. Arts activities are important in educational programmes for nursing and other health care professionals [25,26,27,28] with growing evidence that arts activity has a positive impact on people‘s health and well-being [29,30,31,32]. Art activities could be used as a tool to improve well-being at work [33,34]. Thus, the aim of this study was to investigate the impact of arts activity on the well-being of nursing staff working in a hospital.

## 2. Materials and Methods

The study was carried out during October and December 2014 in a hospital in the city Kaunas, in Lithuania. In total there was a nursing staff of 554, out of which 115 took part in the study. Permission to conduct the study was obtained from the hospital administration, the ethics committee, the nurse managers at relevant departments and the questionnaire was approved by the Kaunas Region Biomedical Research Ethics Committee (number BE-2-50). Participation in the study was voluntary. All respondents received written and verbal information about the aim and content of the study. All persons in intervention and control groups consented to take part in the study and anonymity was ensured. 

There was an open call for participation in the study for persons to be involved in intervention and control groups. An open call was announced in an organized meeting at the hospital for nursing staff, and via the hospital’s communication tools. The criteria for persons to be involved in the intervention and control groups were as follows: nursing staff by their position at work; working in this hospital; ability and availability to participate in the study; no previous participation in painting/silk painting activities experience; agreement to take part in the study. After the completion of the intervention group, the control group was formed of nursing staff that did agree to take part in the study and worked in the same hospital departments as persons in the intervention group. The study organizers offered a total of 60 places for the intervention group to take part in arts activity (silk painting activity). Afterwards, 56 members of nursing staff in the intervention group and 59 members of nursing staff in the control group (who did not participate in silk painting activity) registered.

Silk painting activities were offered once a week with five different timing options, and people in the intervention group could choose the most convenient day and time to attend the silk painting activities. Five groups were formed, four groups had 11 persons and one group had 12 persons. The silk painting activities were not art therapy sessions, but art activities (“arts for health” activities)—without a therapy approach, where nursing staff could experience the arts and engage with the silk painting activity, express their creativity, relax, socialize with colleagues, *etc.* In each group, a professional artist was leading the silk painting activities and was a member of a research team. Each person attended one silk painting activity once a week over a 10-week period. All activities were free of charge and lasted for two hours. The activities were carried out in the participants’ work settings, but outside their regular work hours. Nursing staff attended the silk painting activities regularly; those who were not able to participate on the selected day could join another activity in that same week. In each silk painting activity, nursing staff explored a different silk painting technique and created various artworks. During the 1st activity, participants were introduced to silk painting theory, techniques, materials and experimented with paints. Each participant in 2nd and 3rd activity painted on silk and created a scarf. In 4th and 5th activity, they created a large scale scarf, and in the 6th and 7th activities, they created a painting. In 8th and 10th activity they created a final painting, which later was presented at a public exhibition in the municipal library and in the hospital. Silk painting was chosen for this study because of its flexibility in techniques, because the participants do not need to have a particular knowledge and skill in painting and because results can be achieved quickly. Furthermore, the findings of a pilot study carried out in 2012 [35], had shown the positive impact of silk painting activities on medical staff well-being (*n* = 34) (one weekly silk painting activity over an 8-week period). 

Data was collected using a survey questionnaire which contained questions on socio-demographic data, the Warwick–Edinburgh Mental Well-Being Scale (WEMWBS), the Short Form-36 Health Survey Questionnaire (SF-36), the Reeder Stress Scale (Stress scale) and the Multidimensional Fatigue Inventory (MFI-20) (before and after art activities in both groups). Before the study, to ensure the clarity and appropriateness of the survey questionnaire, a pilot survey with nursing staff at the hospital (*n* = 10) was carried out. All questionnaires were completed anonymously. Nursing staff understood the questionnaire well, with no corrections suggested and the questionnaire was used in this study. 

The WEMWBS [36] has demonstrated good validity and reliability with general, clinical and community populations [35,37,38,39]. The WEMWBS comprises 14 items that relate to an individual’s subjective mental well-being over the previous two weeks. Each of the 14 item responses in WEMWBS are scored from 1 (none of the time) to 5 (all of the time) and an overall scale score is calculated by totalling the 14 individual item scores. The minimum score is 14 (very poor) and the maximum is 70 (very good). In this study the Cronbach’s alpha coefficient for WEMWBS was 0.9 (very good).

The SF-36 [40] has been validated and used as a generic measure of health status in different target groups such as general population, clinical and medical staff [41,42,43,44,45]. SF-36 relates to an individual’s subjective general health over the previous month. The questionnaire is composed of 36 questions that are grouped into eight subscales: Physical functioning, Role limitations (physical problems), Bodily pain, General health, Vitality/Energy, Social functioning, Role limitations (emotional problems), and Emotional well-being. Respondents rate each item question in increasing order (from 1 to 6). Variants of answers for the items questions were converted into standardized points, when 0 = the lowest score (very poor) and 100 = the highest score (very good). In this study the Cronbach’s alpha coefficient for SF-36 was 0.9 (very good).

The Stress scale [46] has been validated and used in different target groups such as general population and medical staff [47,48,49]. The Stress scale comprises seven items that relate to an individual’s psychosocial stress over the previous month. Each of the seven item responses is scored from 1 (strongly agree) to 4 (strongly disagree) and an overall scale score is calculated by totalling the seven individual item scores. The minimum score is 7 (very poor) and the maximum is 28 (very good). In this study the Cronbach’s alpha coefficient for Stress scale was 0.8 (good).

The MFI-20 [50] has been validated and used in different target groups such as general population, clinical and medical staff [51,52,53,54,55]. The MFI-20 comprises five subscales: General fatigue, Physical fatigue, Mental fatigue, Reduced activity, and Reduced motivation. Each subscale includes four items with 5-point Likert scales (1—strongly agree, 5—strongly disagree). Variants of answers for the scale questions were converted into standardized points, when 0 = the lowest score (very good) and 100 = the highest score (very poor, indicating greater fatigue). In this study the Cronbach’s alpha coefficient for MFI-20 was 0.9 (very good).

The survey questionnaires were filled in by all persons in intervention and control groups prior to the first silk painting activity and at the end of the study, before the final silk painting activity. Prior to being given the questionnaires, the intervention and control groups were introduced to the study aims, research content and questions, and the use of the final data. The questionnaire was given to the respondents; the time for filling them in was not limited. In the intervention group there were no unanswered or incomplete questions, while in control group after the study, four questionnaires were incomplete (so 55 questionnaires were included in further data analysis). 

### Data Analysis 

Statistical data analysis was undertaken. The data were tested for normal distribution using the Kolmogorov–Smirnov test, and the null hypothesis about normal distribution was rejected. Statistical data analysis included: descriptive statistics (frequency, percentage, mean, standard deviation).

Non-parametric statistics analysis: the differences in the total scores in SF-36, MFI-20, WEMWBS and Stress subscales/scales between intervention group and control group at baseline/at post-intervention were calculated using a Man Whitney U Test for two independent samples and the differences in the total scores in SF-36, WEMWBS, MFI-20 and Stress subscales/scales between baseline and post-intervention for both intervention group and control group were calculated using a Wilcoxon signed—ranks test for two related samples.

Fisher’s exact test was used to evaluate the statistical significance of the difference between the answers of the questionnaire. The level of significance was set at *p* ≤ 0.05. Cronbach’s Alpha (Cronbach’s Alpha coefficient, α) was computed to examine the internal consistency of the questionnaire—according to George and Mallery, as cited in [56] α ≤ 0.6—not acceptable, 0.7 ≤ α < 0.8 acceptable, 0.8 ≤ α < 0.9 good, α ≥ 0.9—very good. Survey data were processed using the statistical software IBM SPSS Statistics 22 (IBM Corporation, Armonk, NY, USA) and MS Excel 2013. 

## 3. Results

Demographic and social characteristics of intervention (*n =* 56) and control (*n =* 59) groups showed that all persons in intervention and control groups (*n =* 115) were females and the majority of persons in both groups had vocational and higher-non-university education. By position at work the majority of persons in both groups were nurses aged 40 to 54 years with work experience ranging from 21 ≤ years. 

The majority of persons in both groups worked in the surgery department with work experience at this hospital ranging from 21 ≤ years. The majority of the respondents had a full time/more than a full time position at work and worked on a rotating shift. The majority of intervention and control groups stated that their work was often physically exhausting and emotionally difficult (Table 1).

Furthermore, no participant in either group had any experience in silk painting/painting activity prior to the study and had not been practicing any other arts activity and no participant in either group—intervention and control group—was taking part in any other arts activity during the study period.

Before the art activities, the SF-36 General health, Vitality/Energy, Bodily pain, Emotional well-being, MFI-20 General fatigue, Physical fatigue, Reduced activity, Reduced motivation subscales scores were moderate in both intervention and control group. In both groups the results of the Stress scale were poor, with people experiencing stressful situations daily. The results of WEMWBS, SF-36 Role limitations (emotional problems), Role limitations (physical problems), Social functioning and Physical functioning scales were good. 

The intervention group evaluated better the SF-36 Vitality/Energy (*p =* 0.01) and MFI-20 General fatigue (*p =* 0.03) subscales after art activities than before activities. A tendency in intervention group was observed, noting better results on SF-36, MFI-20 Physical fatigue, Reduced motivation, Mental fatigue subscales, Stress scale and WEMWBS after the art activities when compared to the results prior to the art activities. Moreover, after art activities, the result of SF-36 Social functioning subscale in the intervention group was good, when before art activities the result was moderate. 

The result of SF-36 Emotional well-being subscale after the art activities in the intervention group was better in comparison with control group (*p =* 0.00). There was a tendency towards the results of SF-36 General health, Vitality/Energy, Social functioning, Bodily pain, Role limitations (emotional problems) subscales, MF-20, Stress scales and WEMWBS being better than in control group after the participation in art activities. Also after art activities the results of SF-36 Social functioning subscale and WEMWBS in the intervention were good, while moderate in the control group.

**Figure 1:**
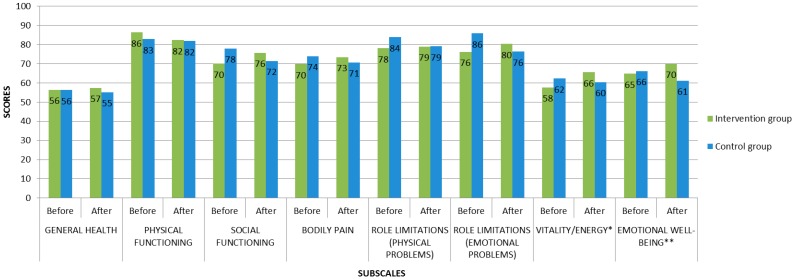
The total scores of SF-36 subscales in intervention and control groups before and after art activities. Note * and **—the difference of the indicators is statistically reliable: *—the differences in the total scores in Vitality/Energy subscale between baseline and post-intervention in the intervention group (*p =* 0.01); **—the differences in the total scores in Emotional well-being subscale between intervention group and control group at post-intervention (*p =* 0.00).

**Figure 2:**
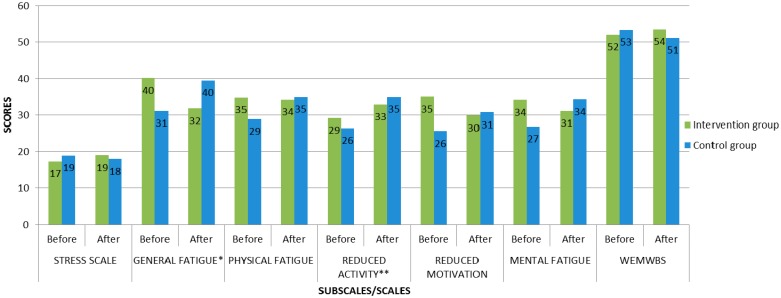
The total scores of MFI-20, Stress scales and WEMWBS in intervention and control groups before and after art activities. Note * and **—the difference of the indicators is statistically reliable: *—the differences in the total scores in General fatigue subscale between baseline and post-intervention in the intervention group (*p =* 0.03); **—the differences in the total scores in Reduced activity subscale between baseline and post-intervention in the control group (*p =* 0.05).

The results of MFI-20 Reduced activity subscale after art activities in the control group were worse than before art activities (*p =* 0.05). And, the results of SF-36, MFI-20, Stress scale and WEMWBS after art activities in the control group tended to be worse than before art activities. After art activities, the results of SF-36 Social functioning subscale and WEMWBS in control group were moderate, while before art activities they were good (Table 2, Table 3 and Table 4) (Figure 1 and Figure 2).

After art activities, the nursing staff in the intervention group felt part of community (*p =* 0.01) and safe (*p =* 0.04) at work, had more energy (*p =* 0.03), were able to concentrate and keep attention better (*p =* 0.02), were able to relax after work (*p =* 0.01) and felt loved (*p =* 0.01) more often than the control group. Furthermore, after the art activities, the intervention group evaluated their health better (*p =* 0.00); felt happier (*p =* 0.04), less nervous (*p =* 0.03), calm and peaceful (*p =* 0.01) and their life was meaningful (*p =* 0.05) more often; resolved their work problems (*p =* 0.02) better and the bodily pain had less of an impact on their general work (*p =* 0.05) than the control group.

After art activities, there was a tendency, within the intervention group, to feel more optimistic about the future, useful, interested in other people, cheerful, confident, interested in new things, the feeling of being more energetic, more relaxed, closer to other people, loved, finding it easier to deal with problems more often than before art activities, in comparison with control group where the results were worse. Moreover, after art activities the results in the intervention group were better than in the control group and after art activities the intervention group felt good about themselves more often than the control group. 

Afterwards, 93% (*n =* 52) of people in the intervention group enjoyed art activities and 75% (*n =* 42) wished to continue silk painting in the future. The majority of the intervention group reported, that the art activities had a positive impact on: reducing work related stress (77%, *n =* 43), improving general health and well-being (68%, *n =* 38), improving mood/sense of happiness (75%, *n =* 42), reducing work related fatigue (physical and mental) (61%, *n =* 34), increasing work productivity (50%, *n =* 28), community building (61%, *n =* 34) with 61% of the intervention group (*n =* 34) wanting to attend various art events more often than they used to. The range of positive emotions experienced by nursing staff in the intervention group were expressed as inspiring, enjoying, exciting, community building, and participants stated they felt happy and relaxed throughout their engagement in arts activity. No negative experiences of the participation in arts activity were stated.

## 4. Discussion 

### 4.1. A Positive Impact on Health and Well-Being

This study focused on a nursing staff that is usually exposed to a variety of health and safety hazards on a daily basis [57,58]. This study revealed that art activities improved nursing staff well-being and general health, and this finding corresponds to other authors’ findings that art activities offer an upstream approach to promoting well-being [35,59,60,61,62] providing public health with a valuable and potentially cost-effective asset-based tool [63,64,65,66]. 

The New Economics Foundation [67] identified the five ways to human well-being: to connect, be active, take notice, keep learning, and to give. According to Aked and Thompson [68], the five ways to well-being are used to motivate behaviour change in individuals across a number of different settings, including work environments, service settings and communities. In this research project, the art activities evidently encouraged personal growth and flourishing, created a positive work environment and provided access to five ways to well-being. This study supports Wright *et al*.’s [69] findings, where the engagement in arts activity embraced the five ways to well-being. 

In this study the participants’ experience of partaking in arts activity could be well described in the model of transformational change analysed by Kilroy *et al*., as cited in [70]. Within a supportive environment and culture, in a period of connection, arts activity generates the possibility of entering a creative flow state, where people typically experienced absorption, deep concentration and engagement in what they were doing. There is a lift in mood and expectations, which opens up perceived possibilities for change; people begin to see things differently and from this a greater sense of well-being arises. In response to this change in state, comes the possibility of a shift or transformation of existing thinking or patterns of behaviour. 

In this study, the intervention group described art activities as inspiring, joyful and exciting, and the participants stated feeling happy throughout their engagement in arts activity. These study findings are closely related to the dynamic model of well-being. According to some authors, when a person is happy, evaluates her/his life as going well and functions positively—she/he can be considered to have a high state of well-being—to be flourishing (Thompson and Marks, [71]). This study also supports the findings of Rollins *et al.* [72], indicating that arts activities create a common, more normative environment, and offer an opportunity for creativity and self-expression. The participation in art activities increased participants’ self-esteem, stimulated personal growth and awakened creativity. After art activities, the intervention group have been feeling good about themselves, confident, interested in new things more often than before art activities, whereas the control group results were worse. Afterwards, 75% of the intervention group indicated a desire to continue silk painting in the future with 61% wanting to attend various art events more often than they used to. 

Maben *et al*. [73], emphasised the importance of investing in and supporting individual staff well-being at work in order to enable staff to better deliver high quality patient care. Sonke *et al.* [74] stated that arts tools can positively affect unit culture, nursing practice, and quality of care on short-stay medical–surgical units. According to Jeffrey *et al*. [6], people who achieve higher standards of well-being at work are likely to be more creative, loyal to their employer, productive, and provide better customer satisfaction. This study data are consistent with the research of Bygren *et al.* [75], where the arts activities for medical care staff (8 weeks totally, once per week) improved perceived Physical health, Social functioning, and Vitality/Energy subscales of SF-36 in the intervention group and decreased among the control group. The study findings support Tuisku *et al.* [76], indicating that arts activities have a positive impact on health care staffs’ well-being and health at work, increasing personal growth and work productivity. The integration of the arts into hospitals has been shown to benefit nurses by increasing both engagement and well-being at work (Wikstrom *et al.* [74]). 

In this study, the participation in art activities improved peoples’ self-assessed health (*p =* 0.00), SF-36 Vitality/Energy (*p =* 0.01), and Emotional well-being (*p =* 0.00). After art activities the bodily pain had less of an impact on their general work (*p =* 0.05) and nursing staff had more energy (*p =* 0.03), when the control group results were worse. There was a tendency for art activities to have a positive impact on the intervention group in SF-36 Social functioning, Physical Health, Role limitations (physical problems), Role limitations (emotional problems) subscales scores and WEMWBS, as after art activities, results were better than before art activities, where the control group evaluated these subscales worse. Afterwards, 68% of the intervention group reported, that the art activities had a positive impact on improving general health and well-being and 50% increasing work productivity.

### 4.2. Increasing Positive Emotions: A Positive Impact on Mental Well-Being 

According to Tuisku *et al.* [76], employees facing job demands and changes at work might benefit from arts when recovering from mental strain, reflecting on their values and finding new perspectives. Theorell *et al.* [33], found a significant cross-sectional linear relationship between artistic activities at work and mental employee health, particularly with emotional exhaustion. This study data is consistent with the findings of Karpavičiūtė and Parkinson [35], where the silk painting activities for medical care staff (eight weeks in total, once per week) had a positive impact on general health and mental well-being, helped to reduce stress at work, increased self-esteem and all participants reported enjoyment. In this study, participation in art activities improved participant’s SF-36 Emotional well-being subscale results (*p =* 0.00) and after art activities the intervention group felt happier (*p =* 0.04) more often compared to the control group. Also after art activities, the intervention group felt more optimistic about the future, relaxed, cheerful, and interested in new things more often than before art activities, where the control group results were worse. 

The World Health Organization has emphasised that there could be a shortage of 12.9 million healthcare workers by 2035 [77]. Almalki *et al.* [23], found a significant association between quality of work life and turnover intention of nurses. The working environments associated with high levels of emotional exhaustion or high job demands take their toll on staff even if staff were performing well [73]. The results of the present study support the statement of Jeffrey *et al* [6], that taking steps to improve relationships at work and encouraging positive feelings can possibly enhance not only job satisfaction, but also life satisfaction. The engagement with arts activities can enhance one’s mood, emotions, and other psychological states as well as reduce depression [65]. After art activities, people in the intervention group felt loved (*p =* 0.01), and stated that their life was meaningful (*p =* 0.05) more often than in the control group. The intervention group described their experience of participation in art activities as enjoying and exciting and that they felt happy throughout their engagement with arts activity. 75% of people reported, that the art activities had a positive impact on improving their mood/sense of happiness.

### 4.3. Reducing Stress

Joslin *et al.* [41], emphasised that managing psychological stress will lead to improved quality of life for nurses, financial benefits for the system, and improved patient care. Arts-based encounters can be effective in reducing stress and burnout in health care workers [78]. The research of Huss and Sarid [79], suggested that it is a timely moment to search for a short term and self-initiated arts based technique of stress reduction that will access health professionals’ creative recourses as an antidote to stress at work. The results of this study support Stuckey and Nobel [65] and confirm, that engagement in art activities can reduce stress and serve as a vehicle for alleviating the burden of chronic disease. Furthermore this study found, that after the art activities, the participants in the intervention group were able to relax after work (*p =* 0.01), they felt less nervous (*p =* 0.03), and were calm and peaceful (*p =* 0.01) more often than the control group. The intervention group described their experience of engagement with arts activity as feeling relaxed and afterwards 77% of nursing staff in the intervention group reported, that the art activities had a positive impact on reducing work related stress. A tendency was observed, that art activities had a positive impact on the Stress scale results in the intervention group and after art activities, they felt relaxed more often than the control group. 

### 4.4. Reducing Fatigue

Fatigue among nurses is related to poor work performance, attentional failures, and injuries [80]. In this research art activities improved the results of MFI-20 General fatigue subscale (*p =* 0.03) in the intervention group, and those in the intervention group were able to concentrate and keep attention better (*p =* 0.02), while in the control group the results were worse. After art activities, the control group evaluated MFI-20 Reduced activity scale (*p =* 0.05) worse than before art activities and the intervention group evaluated this scale better than control group. Afterwards, 61% of the intervention group reported, that the art activities had a positive impact on reducing work related fatigue (physical and mental), and a tendency was observed that art activities had a positive impact on the results of MFI-20 Physical fatigue, Reduced motivation and Mental fatigue subscales, while in the control group the results were worse. 

### 4.5. Increasing Sense of Community at Work 

Maben *et al.* [73], have highlighted the importance of the local work climate for staff well-being and patient care performance. Jensen [61] stated that art participation has the potential to become a catalyst for generating social capital. Other authors [81,82] have stated that social support is a very important factor in the reduction of stress experienced at work, burnout prevention and improvement in quality of life. Gustainienė and Bakšienė [48] emphasized the importance of social support for medical staff well-being. According to Gerikienė [83], there is a need to improve physical and psychological working conditions of nursing staff which would increase self-esteem, create a friendly work environment that increases collaboration and trust among staff. This study found, that art activities had a positive impact on improving communication and relationships and enabled a sense of community at work in the intervention group. After art activities, participants in the intervention group felt they were part of a community (*p =* 0.01), safe (*p =* 0.04) at work more often than participants in the control group, and the intervention group described the art activities as community building. There was a tendency that art activities improved the result of SF-36 Social functioning subscale in the intervention group, and after art activities participants felt more useful, interested in other people and closer to other people more often than before art activities, while in the control group these results were worse. 61% of the intervention group reported, that the art activities had a positive impact on community building. The study findings support the research of Karpavičiūtė and Parkinson [35], where silk painting activities for medical staff had a positive impact on improving communication and relationships, increasing socialization and a sense of community at work.

## 5. Conclusions

This study of participation in creative arts activity using a naturalistic methodology, shows that the results are related to what could be achieved in a daily workplace setting, and could be adopted in cross-cultural and cross-sectorial settings. The WEMWBS, SF-36, MFI-20 and the Stress scale were appropriate instruments for measuring well-being in this research, and could be used in a broader context to explore the arts as a vehicle to promote the well-being of the health care workforce. The study findings suggest, that arts activity as a workplace intervention, can be used to promote nursing staff health and well-being at work, manage occupational stress and health risks at work, and strengthen organizational well-being.

### Limitations of the Study 

The main limitations of this study were a relatively small sample size (the experiences of the intervention group cannot be generalised to represent a larger setting); single gender cohort (all participants were female, but the gender structure of our study reflects the global situation where the majority of nursing staff are female); and a short time frame. Further research might explore the cost effectiveness of participation in art activities; include the evaluation of participants’ health and well-being a few months/years after the study and examine participants’ independent engagement in the arts (such as attendance of cultural events, *etc.*) following the study period and after the study; involve participants from rural areas and different medical backgrounds; explore different art activities and the impact of arts activity according to the participants’ socio-demographic characteristics.

## Figures and Tables

**Table 1:** Demographic and social characteristics of intervention (*n =* 56) and control (*n =* 59) groups.

Characteristics *	Intervention Group *n* (%)	Control Group *n* (%)
**Gender**		
Female	56 (100)	59 (100)
Age in groups (years)		
21–24	7 (12)	2 (3)
25–39	11 (20)	9 (15)
40–54	32 (57)	33 (56)
55–70	6 (11)	15 (25)
Education		
Secondary	9 (16)	7 (12)
Vocational **	16 (29)	30 (51)
Higher non-university	17 (30)	14 (24)
Higher university	14 (25)	8 (13)
Position at work		
Nurse	39 (70)	48 (81)
Nurse assistant	17 (30)	11 (19)
Work experience as nurses/nurse assistants (years)		
1–5	15 (27)	9 (15)
6–10	15 (27)	8 (14)
11–15	3 (5)	5 (8)
16–20	8 (14)	11 (19)
≥21	15 (27)	26 (44)
Work experience at this hospital (years)		
1–5	15 (27)	15 (25)
6–10	14 (25)	12 (20)
11–15	4 (7)	5 (8)
16–20	7 (12)	9 (15)
≥21	16 (29)	18 (31)
Department		
Palliative care	12 (21)	13 (22)
Surgery	19 (34)	18 (31)
Therapeutic	10 (18)	8 (14)
Emergency	6 (11)	9 (15)
Obstetrics	4 (7)	5 (8)
Intensive care	5 (9)	6 (10)
Personal workload in this hospital		
0.6–1.0 full working time	29 (52)	27 (46)
More than 1.1–1.5 full working time	27 (48)	32 (54)
Work shift		
Day	23 (41)	20 (25)
Night	4 (7)	8 (17)
24-h	1 (2)	5 (14)
Rotating	28 (50)	26 (44)
Work is physically exhausting		
Rarely	5 (9)	4 (7)
Sometimes	10 (18)	20 (34)
Often	27 (48)	22 (37)
Always	14 (25)	13 (22)
Work is emotionally difficult		
Rarely	1 (2)	4 (7)
Sometimes	8 (14)	17 (29)
Often	24 (43)	21 (35)
Always	23 (41)	17 (29)

Note *—There were no statistical differences between the groups in demographic and social characteristics, when *p* > 0.05; ** This is nursing education for those that graduated from medical school before 2001. All medical schools became colleges after the education reform, some courses were closed down for good and others were reformed.

**Table 2:** The differences in the total scores in SF-36, MFI-20, WEMWBS and Stress subscales/scales between baseline and post-intervention in the intervention group.

Title of Subscale/Scale	The Differences in the Total Scores in Subscales/Scales between Baseline ** and Post-intervention *** in the Intervention Group		
	Before Art Activities, *n =* 56 Mean (SD ****)	After Art Activities, *n =* 56 Mean (SD ****)	(*p* value)
SF-36 Physical functioning	86 (14)	82 (15)	(0.06)
SF-36 General health	56 (14)	57 (15)	(0.25)
SF-36 Vitality/Energy	58 (15)	66 (14)	*
SF-36 Social functioning	70 (19)	76 (20)	(0.06)
SF-36 Bodily pain	70 (20)	73 (18)	(0.36)
SF-36 Role limitations (physical problems)	78 (27)	79 (33)	(0.74)
SF-36 Role limitations (emotional problems)	76 (28)	80 (29)	(0.51)
SF-36 Emotional wellbeing	65 (15)	70 (15)	(0.14)
MFI-20 General fatigue	40 (21)	32 (22)	(0.03) *
MFI-20 Physical fatigue	35 (23)	34 (23)	(0.58)
MFI-20 Reduced motivation	35 (18)	30 (20)	(0.13)
MFI-20 Mental fatigue	34 (20)	31 (19)	(0.39)
MFI-20 Reduced activity	29 (19)	33 (21)	(0.47)
WEMBWS	52 (7)	54 (7)	(0.15)
Stress scale	17 (4)	19 (4)	(0.06)

Note *—the difference of the indicators is statistically reliable, when *p* ≤ 0.05; **—before art activities; ***—after art activities; ****—the standard deviation.

**Table 3:** The differences in the total scores in SF-36, MFI-20, WEMWBS and Stress subscales/scales between baseline and post–intervention in the control group.

Title of Subscale/Scale	The Differences in the Total Scores in Subscales/Scales between Baseline ** and Post-intervention *** in the Control Group		
	Before Art Activities, *n =* 59 Mean (SD ****)	After Art Activities, *n =* 55 Mean (SD ****)	(*p* value)
SF-36 Physical functioning	83 (19)	82 (18)	(0.98)
SF-36 General health	56 (14)	55 (13)	(0.60)
SF-36 Vitality/Energy	62 (13)	60 (16)	(0.89)
SF-36 Social functioning	78 (18)	72 (21)	(0.17)
SF-36 Bodily pain	74 (23)	71 (20)	(0.44)
SF-36 Role limitations (physical problems)	84 (28)	79 (30)	(0.54)
SF-36 Role limitations (emotional problems)	86 (29)	76 (31)	(0.20)
SF-36 Emotional wellbeing	66 (17)	61 (17)	(0.24)
MFI-20 General fatigue	31 (22)	40 (23)	(0.10)
MFI-20 Physical fatigue	29 (23)	35 (23)	(0.25)
MFI-20 Reduced motivation	26 (19)	31 (18)	(0.18)
MFI-20 Mental fatigue	27 (22)	34 (25)	(0.19)
MFI-20 Reduced activity	26 (22)	35 (25)	(0.05) *
WEMBWS	53 (7)	51 (9)	(0.14)
Stress scale	19 (5)	18 (4)	(0.43)

Note *—the difference of the indicators is statistically reliable, when *p* ≤ 0.05; **—before art activities; ***—after art activities; ****—the standard deviation.

**Table 4:** The differences in the total scores in SF-36, MFI-20, WEMWBS, and Stress subscales/scales between intervention group and control group at post-intervention.

Title of Subscale/Scale	The Differences in the Total Scores in Subscales/Scales between Intervention Group and Control Group at Post–Intervention **		
	Intervention Group, *n =* 56 Mean (SD ***)	Control Group, *n =* 55 Mean (SD ***)	(*p* value)
SF-36 Physical functioning	82 (15)	82 (18)	(0.73)
SF-36 General health	57 (15)	55 (13)	(0.15)
SF-36 Vitality/Energy	66 (14)	60 (16)	(0.07)
SF-36 Social functioning	76 (20)	72 (21)	(0.24)
SF-36 Bodily pain	73 (18)	71 (20)	(0.43)
SF-36 Role limitations (physical problems)	79 (33)	79 (30)	(0.51)
SF-36 Role limitations (emotional problems)	80 (29)	76 (31)	(0.40)
SF-36 Emotional wellbeing	70 (15)	61 (17)	(0.00) *
MFI-20 General fatigue	32 (22)	40 (23)	(0.07)
MFI-20 Physical fatigue	34 (23)	35 (23)	(0.95)
MFI-20 Reduced motivation	30 (20)	31 (18)	(0.65)
MFI-20 Mental fatigue	31 (19)	34 (25)	(0.64)
MFI-20 Reduced activity	33 (21)	35 (25)	(0.67)
WEMBWS	54 (7)	51 (9)	(0.07)
Stress scale	19 (4)	18 (4)	(0.32)

Note *—the difference of the indicators is statistically reliable, when *p* ≤ 0.05; **—after art activities; ***—the standard deviation.

## Abbreviations

WEMWBS	The Warwick-Edinburgh Mental Well-Being Scale
SF-36	The Short Form-36 Health Survey Questionnaire
Stress scale	The Reeder Stress Scale
MFI-20	The Multidimensional Fatigue Inventory
